# Research Advances in the Role of the Poly ADP Ribose Polymerase Family in Cancer

**DOI:** 10.3389/fonc.2021.790967

**Published:** 2021-12-16

**Authors:** Huanhuan Sha, Yujie Gan, Renrui Zou, Jianzhong Wu, Jifeng Feng

**Affiliations:** ^1^ Department of Chemotherapy, Jiangsu Cancer Hospital, Jiangsu Institute of Cancer Research, The Affiliated Cancer Hospital of Nanjing Medical University, Nanjing, China; ^2^ Research Center of Clinical Oncology, Jiangsu Cancer Hospital, Jiangsu Institute of Cancer Research, The Affiliated Cancer Hospital of Nanjing Medical University, Nanjing, China

**Keywords:** PARPs, ADP-ribosylation, cancer, mechanism, biological process

## Abstract

Poly ADP ribose polymerases (PARPs) catalyze the modification of acceptor proteins, DNA, or RNA with ADP-ribose, which plays an important role in maintaining genomic stability and regulating signaling pathways. The rapid development of PARP1/2 inhibitors for the treatment of ovarian and breast cancers has advanced research on other PARP family members for the treatment of cancer. This paper reviews the role of PARP family members (except PARP1/2 and tankyrases) in cancer and the underlying regulatory mechanisms, which will establish a molecular basis for the clinical application of PARPs in the future.

## Introduction

Poly ADP ribose polymerases (PARPs) are a family of proteins with a homologous catalytic domain involved in ADP-ribosylation (1). ADP-ribosylation refers to the transfer of ADP-ribose to target proteins in a nicotinamide adenine dinucleotide (NAD)-dependent manner. The conjugation of a single ADP-ribose unit onto each modified residue is known as MARylation. Proteins with a single ADP-ribose modification can be further ADP-ribosylated, which is called poly-ADP-ribosylation, and some PARPs are capable of adding multiple ADP-ribose units (PARylation) (2, 3). This reversible post-transcriptional modification plays a role in the regulation of various biological processes, such as genomic stability, inflammatory regulation, energy metabolism, apoptosis, and signal transduction (4–6). ADP-ribose can be synthesized and degraded within seconds. The PAR-degrading enzyme poly ADP-ribose glycohydrolase hydrolyzes the glycosidic bonds within PAR (7, 8). ADP-ribosylhydrolase 3 (ARH3) hydrolyzes the bond between the protein and the proximal ADP-ribose conjugated at serine residues (9). In addition, several proteins including ARH1, terminal ADP-ribose protein glycohydrolase, and macrodomain-containing 1 and 2 break the ribose–protein bond at different residues (10–12). Histone PARylation factor 1 (HPF1), a PARP1-accessory factor, confers serine specificity to the PAR attachment (13, 14). DNA and RNA can be ADP-ribosylated as well (15–17). In recent years, the successful development of PARP1/2 inhibitors has provided effective treatment options for ovarian cancer (OC) and breast cancer (BC) (18–21). Moreover, it has caused a great interest in other PARP family members in the field of cancer. PARP3 and PARP9 are overexpressed in different human cancer tissues such as BC, cervical cancer (CC), and diffuse large B-cell lymphoma (DLBCL), whereas PARP7 expression is decreased in cancer tissues (22–26). The biological process of cell proliferation, cell apoptosis, cell migration, and invasion are often involved in these cancers. Thus, PARPs can have various functions by targeting different genes involved in biological processes related to cancer. In this review, we summarize the role and regulatory mechanism of PARP3, PARP4, and PARP6–16 in cancer. PARP1/2 and tankyrases are not addressed because they have been reviewed extensively. The expression patterns and functions of these PARPs suggest that other PARP family members are potential targets in cancer.

## PARP Family Members and Classification

PARP1 is the first identified member of the PARP family. To date, 17 PARP family members have been identified in humans, and they all possess a conserved domain of approximately 230 amino acids. PARP homologues have been found in other animals, plants, fungi, bacteria, and viruses, which indicates that the function of these enzymes may be widely conserved (1, 2). Despite having common domains, they can be classified into four different types according to their special domains or functional characteristics. PARP1 binds to the site of DNA damage and activates the ADP-ribosylation of substrate proteins, which mediate the recruitment of DNA repair factors and the reconstruction of chromosomes around the damaged site (27). PARP2 and PARP3 have similar functions to PARP1, and the presence of PARP3 can promote the damage repair effect of PARP1 (28). Therefore, these three members are classified as DNA-dependent PARPs. PARP5a and PARP5b, which are also known as telomere polymerase 1 (TNKS1) and TNKS2, play an important role in maintaining telomere length and regulating the Wnt and Notch signaling pathways. They belong to the family of tankyrases (29). PARP7, PARP12, and PARP13 have a Cys-Cys-Cys-His zinc-finger domain and are classified as CCCH-type PARPs (30, 31). PARP9, PARP14, and PARP15 are classified as macro-type PARPs because they contain a macro domain consisting of approximately 190 amino acid residues (32, 33). The remaining family members are not included in the above four types because they have different structural characteristics and their functions are not clear. Analysis of self ADP-ribosylation indicates that only PARP1, PARP2, PARP5a, and PARP5b can add multiple ADP-ribose units, whereas the remaining 11 PARPs conjugate a single ADP-ribose to amino acid residues. PARP9 and PARP13 do not have catalytic activity (2). However, when PARP9 is linked to histone E3 ubiquitin ligase 3L (DT3XL), it can catalyze the action of mono ADP-ribosylation (34).

## Role of the PARP Family in BC

PARP inhibitors have achieved satisfactory results in the treatment of BC with BRCA1/2 mutation (35, 36). This approach is based on the concept of synthetic lethality (37). PARP1/2 inhibitors inactivate base excision repair and cause DNA double-strand breaks (DSBs). In BC cells with BRCA1/2 mutations, DSBs are not repaired by homologous recombination (HR), and their accumulation causes cell death (37, 38). In addition, several other PARP members are closely related to the occurrence and development of BC (**Table 1**). Specifically, PARP3 expression is higher in BC cells of the mesenchymal phenotype and promotes stem-like cell properties in BC cells by inducing the expression of stem cell markers of the sex-determination-related gene cluster 2 and octamer binding transcription factor 4 and by promoting stem cell self-renewal (39). The expression of PARP3 is higher in BC tissues than in the control group and significantly associated with histological grade. Moreover, PARP3-overexpressing BC patients, especially BRCA1-positive patients, have shorter disease-free survival (22). Beck et al. showed that PARP3 ADP-ribosylates glycogen synthase kinase-3β, a positive regulator of rapamycin-insensitive companion of TOR (Rictor) ubiquitination and degradation (44, 45). The Rictor/mammalian target of rapamycin complex 2 (Rictor/mTORC2) pathway is involved in cell proliferation, survival, and epithelial-to-mesenchymal transition (EMT) (46). PARP3 selectively suppresses the growth, survival, and *in vivo* tumorigenicity of BRCA1-deficient triple-negative BC (TNBC) cells. Therefore, PARP3 and BRCA1 are synthetic lethal, and targeting the catalytic activity of PARP3 might be a promising therapeutic strategy for BRCA1-associated cancers (44). In addition, PARP3 inhibitors sensitize BC cells to vinorelbine, a drug used in the treatment of metastatic BC. These results indicate that combination treatment with PARP3 inhibitors and vinorelbine is a promising strategy for BC patients with metastases (47).

**Table 1:** Role of PARP family in BC.

Member	Expression/Germline Variants/Status	Biological Process	Clinicopathological Parameters	Reference
PARP3	Higher (cancer cells of mesenchymal-like basal B subtype vs epithelial-like luminal subtypes)	Cell self-renewal	—	(39)
PARP3	Higher (cancer tissues vs tumor-adjacent tissues)	—	Overexpression associated with histological grade II–III, shorter DFS time and exhibited a tendency toward shorter OS	(22)
PARP4	Higher frequency (G496V and T1170I) (cancer participants vs the controls)	Cell proliferation	Low expression associated with poor PFS and OS (GEO, EGA, and TCGA datasets)	(40)
PARP7	Lower (tumor tissues vs normal tissues)	—	Higher expression related to preferable survival (all available databases online at that time)	(25)
PARP7	—	Cell proliferation	Lower expression associated with worse prognosis	(41)
PARP9	Higher (ER^+^-tumor tissues vs tumor-adjacent tissues)	—	—	(42)
PARP9	Higher (cancer tissues vs paired normal breast tissues)	Cell migration	Overexpression negatively associated with ER expression, positively associated with axillary lymph node metastasis	(43)

BC, breast cancer; DFS, disease-free survival; OS, overall survival; PFS, progression-free survival; GEO, Gene Expression Omnibus; EGA, European Genome-phenome Archive; TCGA, The Cancer Genome Atlas; ER, estrogen receptor.

Whole-exome sequencing showed that the frequencies of the PARP4 variants, G496V and T1170I, are significantly higher in BC tissues. Survival analysis shows that low expression of PARP4 is correlated with poor progression-free survival (PFS) and overall survival (OS), suggesting that PARP4 functions as a tumor suppressor in BC (40). However, the results of functional analyses do not support the role of PARP4 as a susceptibility gene, emphasizing the importance of functional analysis for verifying candidate cancer susceptibility genes (48). In BC cells, PARP6 regulates the activity of cycle checkpoint kinase 1 (Chk1), a key downstream factor in the ataxia telangiectasia and Rad3-related (ATR) pathway (49), *via* ADP-ribosylation, thereby maintaining centrosome integrity and ultimately promoting the development of BC (50, 51). PARP7 expression is lower in BC tissues than in normal tissues, and its downregulation is associated with worse prognosis (25, 41). Recent studies suggest that estrogen receptor α (ERα), the dominant regulator of estrogen action in breast tissue, regulates PARP7 expression and that PARP7 acts as a negative regulator of ERα activity *via* mono-ADP-ribosylation in BC cells (52, 53). By contrast, PARP9 expression is higher in BC than in paired normal breast tissues, and its expression is positively correlated with axillary lymph node metastasis and negatively associated with ER expression. These data suggest that PARP9 promotes the progression of BC (42, 43). In addition to the above PARP family members, PARP13 is also involved in mammary tumorigenesis. Heat shock transcription factor 1 recruits PARP1 through PARP13. The ternary complex formation protects cells from DNA damage by promoting DNA repair, and supports the growth of BRCA1-null mammary tumors (54).

## Role of the PARP Family in Malignancies of the Digestive System

### Role of the PARP Family in Esophageal Cancer and Gastric Cancer

The PARP family plays an important role in gastrointestinal tumors (**Table 2**). Single‐cell intratumoral stemness analysis revealed that PARP4 is a cancer stemness-associated gene in esophageal squamous cell carcinoma (ESCC). In a cohort of 121 patients with ESCC, immunohistochemical scoring indicated that high PARP4 expression is associated with poorer survival (65). The expression of PARP9 is upregulated in EC tissues and EC cells and may be regulated by circular RNA PRKCI (circPRKCI), affecting the viability, colony formation, cell cycle progression, and radiosensitivity of EC cells (55). In GC, PARP6 expression is higher in cancer tissues and cells than in normal gastric mucosa tissues and cells. PARP6 promotes cell proliferation, migration, and invasion of GC cells partly by activating survivin (56), a member of the inhibitor of apoptosis protein family (66). Analysis of an online database indicated that PARP10 expression increases survival in GC and showed that PARP10 is involved in the regulation of fatty acid degradation, promoting further studies to understand of the role of PARP10 in metabolic regulation (57). Most of these studies focused on the relationship between PARP expression and clinical characteristics, and few studies performed functional analyses. Further studies are needed to clarify the roles of these PARPs in EC and GC in the future.

**Table 2:** Role of PARP family in digestive system tumor.

Cancer	Member	Expression/Germline Variants/Status	Biological Process	Clinicopathological Parameters	Reference
EC	PARP9	—	Cell cycle	—	(55)
GC	PARP6	Higher (cancer tissues and cells vs normal gastric mucosa tissues and cells)	Cell proliferation, migration, and invasion	—	(56)
	PARP10	—	—	Low expression associated with longer survival (online database kmplot.com)	(57)
HCC	PARP6	Lower (tumor tissues vs tumor-adjacent tissues)	Cell proliferation, migration, and invasion	Expression level negatively associated with clinical stage, TNM stage, and metastasis	(58)
	PARP10	Lower (tumor tissues vs tumor-adjacent tissues)	Cell proliferation	Low expression associated with poor OS and DFS	(59)
HB	PARP6	Methylation	—	Associated with poorer OS and poorer EFS	(60)
PC	PARP14	Higher (tumor tissues vs tumor-adjacent tissues)	Cell proliferation and apoptosis	Higher expression associated with poor OS	(61)
CRC	PARP6	Higher (adenocarcinoma tissues and cancer cells vs adjacent non-tumor tissues and a normal colon cell line)	Cell cycle, apoptosis, and invasion	—	(62)
	PARP6	—	Cell proliferation, apoptosis, migration, and invasion	Higher expression associated with higher OS rate	(63)
	PARP6	—	Cell proliferation and cycle	Positivity inversely associated with loss of histological differentiation; PARP6-positive associated with a good prognosis	(64)

EC, esophageal cancer; GC, gastric carcinoma; HCC, hepatocellular carcinoma; TNM, Tumor, Node, Metastasis; OS, overall survival; DFS, disease-free survival; HB, hepatoblastoma; EFS, event-free survival; PC, pancreatic cancer; CRC, colorectal cancer.

### Role of the PARP Family in Hepatic Tumors and Pancreatic Cancer

**Figure 1:**
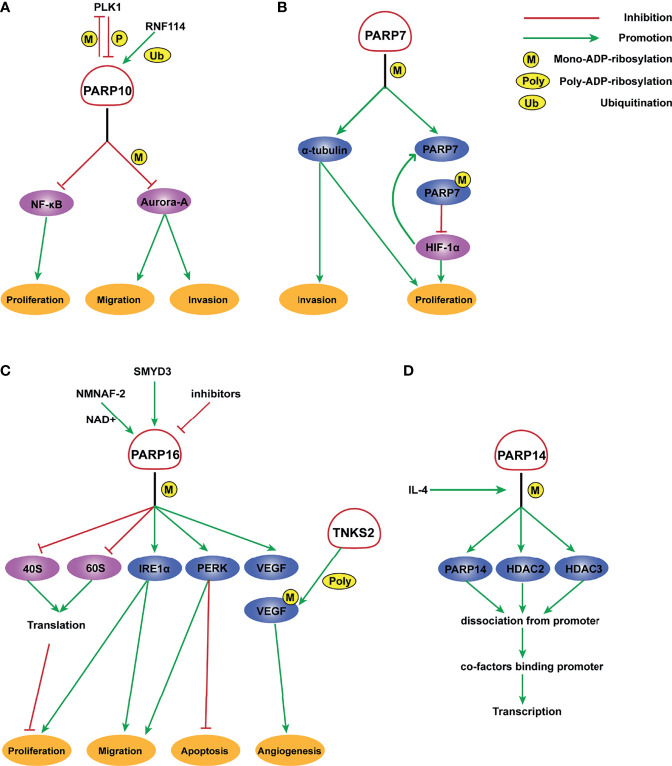
Molecular mechanism of PARP7, PARP10, PARP14, and PARP16 ADP-ribosylation. **(A)** PLK1 phosphorylates PARP10 and disrupts its inhibition of NEMO ubiquitination, thereby enhancing the transcriptional activity of NF- κB. In turn, PARP10 mono-ADP-ribosylates PLK1, whose kinase activity and oncogenic function are significantly inhibited by MARylation. RNF114 promotes the ubiquitination of PARP10, and PARP10 mono-ADP-ribosylates Aurora-A and inhibits its kinase activity, thereby playing an important role in tumor proliferation and metastasis suppression. **(B)** PARP-7 MARylates α-tubulin to promote microtubule instability, which may regulate cancer cell growth and motility. HIF-1 promotes the transcription of PARP7, which serves to deactivate HIF-1 in an ADP ribosylation-dependent manner. **(C)** A potential inhibitor of PARP16 suppresses the ER stress-induced phosphorylation of PERK and IRE1α, thereby increasing cancer cell apoptosis. SMYD3 can bind to the promoter of PARP16 and activate its transcription, increasing cell proliferation and invasion. NMNAT-2 supports the catalytic activity of PARP16, which MARylates ribosomal proteins. Ribosome MARylation promotes protein homeostasis in cancers by fine-tuning the levels of protein synthesis and preventing toxic protein aggregation. In addition, PARP16 mono ADP-ribosylates VEGF, which is further poly ADP-ribosylated by TNKS-2, leading to the activation of downstream pathways that promote angiogenesis. **(D)** PARP14 mono-ADP-ribosylates HDAC2, HDAC3, and itself with IL-4 occurrence, which facilitates the dissociation of PARP14 and HDAC complex from the promoter, leading to the binding of transcription co-factors to the promoter and initiating gene transcription.

The role of PARP6 in hepatocellular carcinoma (HCC) cells is inconsistent with that in GC. It is expressed at a low level in tumor tissues and is negatively correlated with clinical stage in HCC. Moreover, PARP6 inhibits the expression of X-ray repair cross complementing 6 (XRCC6) by inducing its degradation, thereby affecting the Wnt/β-catenin signaling pathway and ultimately inhibiting the occurrence and development of HCC (58). PARP10 delays the progression of HCC (**Figure 1A**). Zhao et al. demonstrated that PARP10 expression is lower in metastatic cancer tissues than in primary cancer tissues and adjacent tissues (67). Aurora-A is a serine/threonine kinase that is overexpressed in multiple human cancers and involved in tumor cell migration and invasion (68, 69). PARP10 inhibits the kinase activity of Aurora-A by ADP-ribosylation, thereby regulating its downstream signals related to metastasis (67). Furthermore, PARP10 phosphorylation at T601 by polo-like kinase 1 (PLK1) suppresses its inhibition of nuclear factor kappa-B (NF-κB) essential modulator (NEMO) ubiquitination, which increases the transcriptional activity of NF-κB toward multiple target genes. PLK1 is mono-ADP-ribosylated by PARP10, which inhibits its kinase activity and oncogenic function in HCC (59). These findings identify a PLK1/PARP10/NF-κB signaling axis and suggest potential therapeutics for PARP10-expressing HCC. PARP12 also acts as a cancer suppressor in HCC by regulating the stability of four-and-a-half LIM-only protein 2 (FHL2) (70). FHL2 is a negative regulator of transforming growth factor β1 (TGF-β1), a potent EMT driver that plays critical roles in the EMT process (71–73). Therefore, PARP12 suppresses HCC metastasis by negatively regulating TGF-β1 expression *via* FHL2 (70). By contrast, PARP14 promotes the development of HCC by inhibiting c-Jun N-terminal kinase 1 (JNK1), which phosphorylates and activates pyruvate kinase M2 (PKM2), thus maintaining low activity of PKM2 and promoting aerobic glycolysis in HCC (74). This study provided a mechanistic link between PARP14, cancer cell apoptosis, and metabolism.

Hepatoblastoma (HB) is very rare but the most common malignant neoplasm of the liver. PARP6 is methylated in HB and significantly associated with poor OS, suggesting that it could be a useful molecular marker to predict a poor outcome in HB patients (60). PC has one of the worst prognoses among different cancer types. The PARP inhibitor olaparib is associated with a longer PFS in patients with advanced PC with BRCA1/2 mutation (75). In addition, PARP4 and PARP14 might play a significant role in PC as well. Alimirzaie et al. suggested that PARP4 is a potential candidate gene for susceptibility to PC (76). PARP14 is highly expressed in primary PC specimens and significantly associated with poor prognosis. PARP14 promotes cell proliferation and gemcitabine resistance in PC cells through the NF-κB signaling pathway, indicating its potential role as a therapeutic target for PC (61).

### Role of the PARP Family in Colorectal Cancer and Colorectal Neuroendocrine Tumors

Several PARP family members play a role in CRC. A gene set meta-analysis identified PARP4 as a candidate prognostic factor for CRC metastasis (77). In addition, the PARP4^V458I^ mutation is only detected in patients with liver metastasis and results in dysregulated protein abundance (78). Proteogenomics analysis showed that PARP4 copy number is markedly increased in CRC (79). Colorectal NETs are rare neoplasms, and Wang et al. found two missense mutations (rs77269056 and rs73172125) in the PARP4 gene, suggesting that PARP4 is a candidate gene involved in colorectal NETs (80). The results of these exploratory analyses need to be confirmed, and functional experiments demonstrating the role PARP4 in CRC or in colorectal NETs need to be performed.

The role of PARP6 in CRC is controversial. PARP6 expression is higher in well-differentiated CRC tissues, and PARP6 positivity is negatively correlated with the expression of Ki-67, a proliferation index. Survival analyses show that PARP6-positive patients have a better prognosis. Based on these results, Tuncel et al. proposed that PARP6 might be a cancer suppressor (64). Furthermore, PARP6 plays an inhibitory role by downregulating the expression of survivin in CRC (63). However, another study showed that the expression levels of PARP6 and survivin are higher in colorectal adenocarcinoma tissues and cells than in adjacent tissues and normal intestinal mucosal cells. PARP6 downregulation inhibits cell survival and invasion and induces G0/G1 arrest and apoptosis (62). These contradictory results could be attributed to the use of samples from different stages or different cell models. The role of PARP6 in CRC remains controversial and needs further investigation.

PARP7 and PARP10 are also involved in CRC. Stable knockdown of PARP7 caused the CRC cell line HCT116 to proliferate significantly faster than control cells. PARP7 downregulates hypoxia-inducible factor 1α (HIF-1α), which is crucial for the adaptive responses of tumors to changes in oxygenation and is often overexpressed in cancers in an ADP-ribosylation dependent manner (41) (**Figure 1B**). PARP10 shows an inconsistent effect on CRC cell lines. Inhibition of the enzymatic activity of PARP10 led to cell cycle arrest accompanied by a marked decrease in proliferative ability (81). Taken together, these results support the importance of these two PARPs in CRC.

## Role of the PARP Family in Urogenital Neoplasms

PARP inhibitors have markedly changed the treatment of OC (82–84). In addition to PARP1/2, other PARP molecules play important roles in OC. Tumors from high-grade serous ovarian cancer (HGSOC) patients who are platinum-resistant have a distinct molecular profile characterized by increased expression of PARP4. This indicates that PARP4 may be a candidate actionable target for platinum-resistant HGSOC (85). However, similar to CRC or colorectal NETs, the role of PARP4 in OC has not been validated with strong experimental evidence. A genome-wide association study suggested a functional role for PARP7 in OC development. PARP7 is markedly downregulated in OC cells compared with primary human ovarian surface epithelial (POE) cells. Similarly, the level of PARP7 expression is lower in OC tissues than in ovary normal tissues (86) (**Table 3**). Goode et al. found that PARP7 MARylates α-tubulin to promote microtubule instability, thereby contributing to several cancer-related biological endpoints such as growth and motility of OC cells although PARP7 mRNA levels are lower in OC patient samples than in non-cancerous tissue (26) (**Figure 1B**). This study identified a large number of PARP7 protein substrates, which may stimulate efforts to expand studies of this PARP molecule. Unlike PARP7, PARP10 expression is upregulated in OC patients (**Table 3**), with 42% of the cohort showing platinum sensitivity and 9% showing resistance (87). This suggests a possible role for PARP10 as a predictor of the response to platinum-based agents. PARP16 also affects OC cell proliferation. The catalytic activity of PARP16 is supported by nicotinamide mononucleotide adenylyl-transferase 2 (NMNAT-2), which is highly upregulated in OC. Depletion of NMNAT-2 or PARP16 decreases the growth of OC cells (88) (**Figure 1C**).

**Table 3:** Role of PARP family in gynecological tumor.

Cancer	Member	Expression/Germline Variants/Status	Biological Process	Clinicopathological Parameters	Reference
OC	PARP7	Lower (cancer tissues from TCGA vs ovary normal tissues from GTEx)	Cell proliferation and invasion	—	(26)
	PARP7	Lower (cancer cells vs POE cells)	—	—	(86)
	PARP10	Higher (cancer tissues vs normal tissues from TCGA)	—	Expression associated with sensitivity to carboplatin and rucaparib	(87)
CC	PARP9	Higher (cancer tissues vs normal tissues)	Cell proliferation and invasion	—	(23)

OC, ovarian cancer; TCGA, The Cancer Genome Atlas; GTEx, Genotype-Tissue Expression; POE, primary human ovarian surface epithelial; CC, cervical cancer.

PARP family members also play a role in CC and prostate cancer. PARP9 expression is higher in CC patients than in controls (**Table 3**). The long non-coding RNA small nucleolar RNA host gene 16 (SNHG16) recruits SPI1 to promote transcription of PARP9 in CC cells. PARP9 downregulation inhibits proliferation and invasion of CC cells (23). Schleicher et al. found that PARP10 promotes cell proliferation, and its overexpression alleviates cellular sensitivity to replication stress. Loss of PARP10 decreases the tumorigenic activity of HeLa cells (89). A recent study demonstrated that PARP10 is regulated by RNF114 and functions as a tumor metastasis suppressor in CC cells (90) (**Figure 1A**). ADP-ribosylation of PARP10 is decreased in RNF114-knockout cells, accompanied by a decrease of Aurora A, a substrate involved in HCC (67). This suggests that RNF114 regulates PARP10 activity, which might in turn inhibit Aurora A activity and downstream signaling. PARP13 acts as a key regulator of the cellular response to tumor necrosis factor–related apoptosis-inducing ligand (TRAIL), a pro-apoptotic cytokine (91). TRAIL binds to its receptors TRAILR1–4. TRAIL binding to TRAILR1 and TRAILR2 triggers the extrinsic apoptotic pathway, whereas TRAILR3 and TRAILR4 act as pro-survival decoy receptors (92–96). PARP13 destabilizes TRAILR4 mRNA post-transcriptionally by binding to its 3′ untranslated region, although it has no effect on the levels of TRAILR1–3. Therefore, PARP13 shifts the balance in the TRAIL signaling pathway towards decreased anti-apoptotic signaling and increased cell sensitivity to TRAIL-mediated apoptosis in HeLa cells (91). PARP16 regulates the unfolded protein response (UPR) of the endoplasmic reticulum (ER). UPR is an ER stress-sensing/repair pathway involved in cell survival and tumor progression (97–99). Inhibition of PARP16 suppresses UPR-related genes, resulting in a dramatic increase of HeLa cell apoptosis (**Figure 1C**). A combination of a PARP16 inhibitor and ER stress-inducing agents might represent a novel approach for cancer therapy (100). Furthermore, PARP16 is a target gene for SET and MYND domain containing 3 (SMYD3), a lysine methyltransferase, and SMYD3 may bind to the promoter of PARP16 to activate host gene transcription (101) (**Figure 1C**). ER-associated PARP16 mono ADP-ribosylates vascular endothelial growth factor (VEGF), the major cytokine regulating angiogenesis, and mono ADP-ribosylated VEGF is poly ADP-ribosylated by Golgi TNKS-2 in HeLa cells, finally leading to angiogenesis (102) (**Figure 1C**). This finding provides evidence of the interplay between PARPs in different organelles.

In metastatic prostate cancer cells, PARP9 and PARP14 were identified as novel oncogenic survival factors. Mechanistically, DTX3L forms a complex with PARP9 and PARP14 and mediates the proliferation as well as drug resistance of prostate cancer cells. Moreover, it was demonstrated for the first time that the enzymatic activity of PARP14 is necessary for the survival of prostate cancer cells (103). Therefore, combined targeted inhibition of PARP14, PARP9, and/or DTX3L might represent a novel therapeutic approach.

## Role of the PARP Family in Malignancies of Hematologic and Lymphatic Systems

PARP9, also termed BAL1 (B-aggressive lymphoma), is expressed at significantly higher levels in fatal high-risk diffuse large B-cell lymphoma (DLBCL) than in low-risk tumors (**Table 4**). PARP9 promotes malignant B-cell migration (24) and is constitutively expressed in a subset of high-risk DLBCLs with an active host inflammatory response. Interferon (IFN) induces PARP9 expression in DLBCL cell lines, and PARP9 is involved in IFN-related signaling pathways, as indicated by the effect of doxycycline-induced BAL1 on upregulating multiple IFN-stimulated genes (109). Although PARP9 has no ADP-ribosylation activity, it interacts with signal transducer and activator of transcription 1 (STAT1) through its macro domains in an ADP-ribosylation–dependent manner. As a consequence, PARP9 mediates proliferation, survival, and chemoresistance in DLBCL by repressing the anti-proliferative and pro-apoptotic IFN-STAT1-IRF1-p53 axis (110). PARP14 and PARP15 also belong to the BAL family and have an effect on lymphoma (33). The effect of interleukin 4 (IL-4) on increasing glycolysis in B cells requires PARP14. Suppression of apoptosis is central to B-lymphoid oncogenesis, and elevated macro-PARP expression is correlated with lymphoma aggressiveness (111). Furthermore, PARP14 alters IL-4 and STAT6-dependent transcription, which plays an important role in B cell responses. In the presence of IL-4, PARP14 promotes efficient ADP-ribosylation of histone deacetylases (HDACs) and itself, leading to the release of HDACs from the promoter to activate transcription (112) (**Figure 1D**).

**Table 4:** Role of PARP family in malignancies of hematologic and lymphatic systems and in other tumors.

Cancer	Member	Expression/Germline Variants/Status	Biological Process	Clinicopathological Parameters	Reference
DLBCL	PARP9	Higher (high-risk tumors vs low-risk tumors)	Cell migration	—	(24)
AML	PARP9	Higher (cancer tissues from TCGA vs normal tissues from GTEx)	—	—	(104)
	PARP15	rs6793271, rs17208928	—	Two polymorphisms associated with increased OS	(105)
MM	PARP14	Higher (cancer cells vs normal B cells)	Cell apoptosis	High expression associated with disease progression and poor survival	(106)
LUAD	PARP15	—	—	Expression associated with better prognosis	(107)
GBM	PARP3	Higher (tumor tissues vs tumor-adjacent tissues)	Cell proliferation	High expression associated with cell radioresistance	(108)

DLBCL, diffuse large B-cell lymphoma; AML, acute myeloid leukemia; TCGA, The Cancer Genome Atlas; GTEx, Genotype-Tissue Expression; OS, overall survival; MM, multiple myeloma; LUAD, lung adenocarcinoma; GBM, glioblastomas.

High expression of PARP9 is associated with poor survival in acute myeloid leukemia (AML) patients (**Table 4**) (104). In addition, two polymorphisms of PARP15 are associated with increased OS in AML (105), suggesting that PARP15 is a potential therapeutic target in AML. The development of tyrosine kinase inhibitors (TKI) has greatly improved the treatment of chronic myeloid leukemia (CML). Islam et al. performed whole-exome sequencing of DNA to reveal genetic variants, which were potential markers for predicting the prognosis of CML patients after TKI discontinuation. PARP9 was one of the markers identified, and its sensitivity, specificity, and positive as well as negative predictive values were verified (113). In multiple myeloma (MM) (**Table 4**), Barbareulo showed that c-Jun N-terminal kinase 2 (JNK2) constitutively suppresses JNK1-mediated apoptosis by affecting expression of PARP14. Moreover, inhibition of PARP14 enhances the sensitization of MM cells to anti-myeloma agents. These authors also found that PARP14 is highly expressed in myeloma plasma cells and associated with poor survival (106). These findings identify PARP14 as a potential therapeutic target in MM. In fact, targeting PARP14 has been proposed as a possible therapeutic approach for not only MM but also multiple cancer types, and PARP14 inhibitors are currently being developed (114). In addition to the aforementioned mechanisms, PARP14 was previously reported to be essential for genomic stability by promoting HR and alleviating replication stress through the regulation of an essential HR factor, RAD51 (115). Subsequently, Moldovan et al. also found that the ATR-Chk1 pathway is essential for the viability of PARP14-deficient cells. PARP14-deficient cells are hypersensitive to both genetic depletion and pharmacological inhibition of this pathway, which is similar to the concept of “synthetic lethality” of existing PARP inhibitors (116). The association between Chk1 and PARP6 has been reported in BC as well (50), highlighting the importance of the ATR-Chk1 pathway and providing further evidence supporting PARPs as potential therapeutic targets in cancer.

## Role of the PARP Family in Other Cancers

Telomere function and DNA damage response pathways are frequently inactivated in cancer. An analysis of 83 non-small-cell lung cancer (NSCLC) tissues and the corresponding control samples indicated that telomere attrition is associated with poor clinical outcomes. Tumors with reactivated telomerase are characterized by downregulation of genes related to DNA repair such as PARP3 (117). This suggests that the relationship between telomerase activity and the loss of several DNA repair genes is involved in the pathogenesis of NSCLC. Additionally, PARP15 is a tumor-infiltrating B lymphocyte-specific gene (TILBSig) that is highly associated with OS (**Table 4**). TILBSigs serve as excellent predictors of the response to immunotherapy and radiotherapy in lung adenocarcinoma patients (107). PARP7 MARylates TANK binding kinase 1, a major kinase related to the activation of the type I IFN response and antiviral immunity, thereby repressing the type I IFN response. Cancer cells may use PARP7 as a mechanism to evade the host immune system by suppressing the type I IFN response, thereby validating T cell-mediated antitumor immunity (118, 119). A recent study showed that oral administration of the PARP7 small-molecule inhibitor RBN-2397 induces antitumor immunity and causes complete tumor regression in a lung cancer xenograft by increasing IFN signaling; a phase 1 clinical trial of RBN-2397 is underway (120, 121). These studies indicate that PARP7 is an important contributor to cancer development. PARP3 is upregulated in primary glioblastoma (GBM) (**Table 4**). Inhibition of PARP3 expression decreases the proliferation of GBM cells and has a synergistic sensitization effect in combination with radiotherapy by interacting with forkhead box M1, enhancing its transcriptional activity (108). Studies in different cancer types suggest the important roles of some PARP family members, although additional studies are needed.

## Conclusion and Prospects

Compared with PARP1/2, other members of the PARP family have been investigated in a limited number of studies; however, their importance in cancer has attracted increased attention in recent years. Studies have highlighted the crucial role of PARPs in the occurrence and progression of human cancer. Dysregulation of PARPs has been demonstrated in a wide range of cancer types, and their differential expression indicates their dual regulatory effects on human cancer. PARP3, PARP9, and PARP14 are usually overexpressed in tumors, whereas PARP7 is normally downregulated. PARP6 and PARP10 have both promoting and suppressive effects on cancer development. Mechanistically, they are widely involved in multiple biological processes, including cell proliferation, apoptosis, migration, and invasion. In addition, they participate in the response of cancers to both chemo- and radiotherapy.

Given the crucial function of PARPs in cancer initiation and progression, therapies targeting PARPs might be promising. However, larger scale studies are needed, as some of the studies focus on clinical relevance, and few have performed functional assays on cells or animal models. Although the biological functions of some PARPs have been validated, the mechanisms underlying the effects often remain unclear. Furthermore, the subcellular localization, biological function, and mechanism of some members of the PARP family such as PARP8 remain unknown in cancer. Additional comprehensive studies are needed to fully elucidate the function of each member of this family.

In conclusion, PARPs are involved a variety of cancer biological characteristics, suggesting their potential in cancer diagnosis, prognosis, and treatment. Nevertheless, extensive research is still needed before the application of PARP-based diagnostic and therapeutic strategies to the clinic.

## Author Contributions

All authors participated in the discussion of the draft. HS and YG: Writing—original draft. RZ and JW: Validation and writing—revision and editing. JF: Conceptualization, supervision, and project administration. All authors contributed to the article and approved the submitted version.

## Conflict of Interest

The authors declare that the research was conducted in the absence of any commercial or financial relationships that could be construed as a potential conflict of interest.

## Publisher’s Note

All claims expressed in this article are solely those of the authors and do not necessarily represent those of their affiliated organizations, or those of the publisher, the editors and the reviewers. Any product that may be evaluated in this article, or claim that may be made by its manufacturer, is not guaranteed or endorsed by the publisher.
